# Dynamics of Replication Fork Progression Following Helicase–Polymerase Uncoupling in Eukaryotes

**DOI:** 10.1016/j.jmb.2019.03.011

**Published:** 2019-05-03

**Authors:** Martin R.G. Taylor, Joseph T.P. Yeeles

**Affiliations:** Division of Protein and Nucleic Acid Chemistry, Medical Research Council Laboratory of Molecular Biology, Francis Crick Avenue, Cambridge, CB2 0QH, UK

**Keywords:** AB, abasic site, CMG, Cdc45–MCM–GINS, CPD, cyclobutane pyrimidine dimer, DDT, DNA damage tolerance, ssDNA, single-stranded DNA, THF, tetrahydrofuran, DNA replication, DNA damage response, DNA repair, replication fork, stalled fork

## Abstract

Leading-strand polymerase stalling at DNA damage impairs replication fork progression. Using biochemical approaches, we show this arises due to both slower template unwinding following helicase–polymerase uncoupling and establishment of prolonged stalled fork structures. Fork slowing and stalling occur at structurally distinct lesions, are always associated with continued lagging-strand synthesis, are observed when either Pol ε or Pol δ stalls at leading-strand damage, and do not require specific helicase–polymerase coupling factors. Hence, the key trigger for these replisome-intrinsic responses is cessation of leading-strand polymerization, revealing this as a crucial driver of normal replication fork rates. We propose that this helps balance the need for sufficient uncoupling to activate the DNA replication checkpoint with excessive destabilizing single-stranded DNA exposure in eukaryotes.

## Highlights

• The replisome displays indistinguishable responses to structurally distinct lesions.
• Cessation of leading-strand synthesis triggers an inbuilt brake in the replisome.
• Fork slowing occurs even with the minimal proteins needed for replication *in vitro*.
• Either Pol ε or Pol δ stalling on the leading-strand impedes fork progression.
• Lagging-strand synthesis always occurs efficiently beyond leading-strand damage.

## Graphical Abstract

**Figure:**
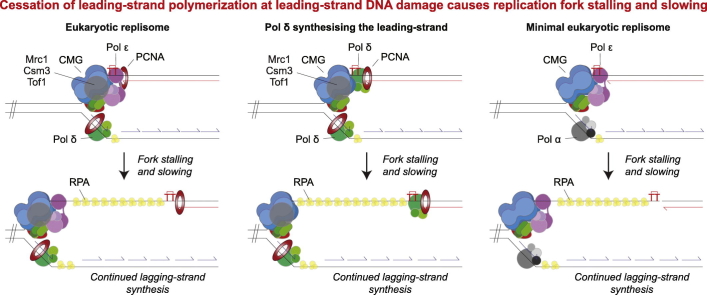

## Introduction

Replication forks often encounter template DNA damage that can stall replicative DNA polymerases. These encounters are thought to slow and/or stall replication fork progression because slow-moving forks and stalled fork structures have been observed in yeast following treatment with UV irradiation or methyl methanesulfonate [1], [2]. Interestingly, in both instances, fork slowing occurred independently of the DNA replication checkpoint, a pathway important for maintaining genome stability during replication stress in eukaryotes, indicating that it was an inherent property of replication through a damaged template. The mechanisms underlying fork slowing and continued fork progression remain to be fully elucidated, but may reflect contributions from replisome-intrinsic features, such as the initial response of the replisome to the polymerization blocking lesion [3], and/or DNA damage tolerance (DDT) pathways, including translesion synthesis and template switching, that have been proposed to engage with forks to help bypass damage [4], [5], [6].

Although fork slowing and stalling occur independently of checkpoint activation, checkpoint kinases have essential roles in stabilizing replication forks to prevent catastrophic replication fork collapse [2]. Checkpoint activation requires both the production of RPA-coated single-stranded DNA (ssDNA) together with continued primer synthesis at replication forks [7], [8], [9], [10]. These criteria can be fulfilled when leading-strand replication is blocked, for example by a DNA lesion, but template unwinding by the replicative CMG (Cdc45–MCM–GINS) helicase and lagging-strand synthesis continue [1], [3]. Intriguingly, although such uncoupled fork progression is a prerequisite for checkpoint activation, excessive uncoupling is deleterious and can lead to RPA exhaustion and the exposure of unstable ssDNA [11], [12]. Accordingly, one function of the replication checkpoint is to restrict uncoupled fork progression following dNTP depletion by hydroxyurea [13], [14]. However, this may not be a generalizable response because extensive uncoupling has been observed in nucleotide excision repair defective but checkpoint proficient yeast exposed to high UV doses [1]. How a balance is struck between sufficient uncoupling to mount a robust checkpoint response and rapid, excessive, and destabilizing uncoupled fork progression remains to be determined. This is in part because our understanding of the intrinsic, early responses of a eukaryotic replisome to DNA damage remains incomplete.

We recently reported some aspects of this initial response by reconstituting collisions between a yeast replisome, assembled with purified proteins, and a cyclobutane pyrimidine dimer (CPD; a common lesion induced by UV irradiation) in the leading- or lagging-strand template [3]. A lagging-strand CPD was rapidly and efficiently bypassed by the replisome leaving a short unreplicated ssDNA gap. By contrast, leading-strand polymerase stalling significantly delayed replication fork progression and was associated with the formation of Y-shaped “stalled forks.” The precise architecture of these stalled forks is yet to be determined. Our work also revealed that the core yeast replisome can restart leading-strand replication beyond a CPD by a leading-strand re-priming mechanism. However, restart by this pathway occurred only at a small subset of stalled forks because leading-strand priming by Pol α is inefficient. Consequently, the majority of replisomes that reached the end of the linear templates (several kilobases beyond the damage) did so having performed extensive template unwinding and lagging-strand synthesis, uncoupled from leading-strand polymerization. This system therefore provides a platform to interrogate the mechanisms and dynamics of uncoupled fork progression beyond leading-strand template damage intrinsic to the replisome. In this work, we have examined factors that influence replication fork stalling and subsequent uncoupled fork progression.

## Results and Discussion

### A generalized response of the yeast replisome to leading-strand template damage

**Fig. 1:**
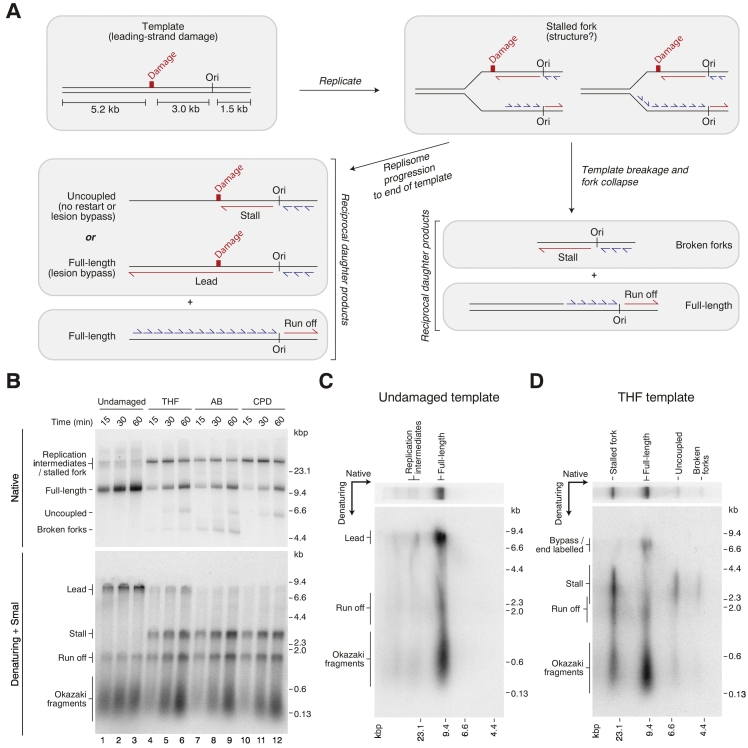
Replisome responses to leading-strand polymerase stalling at different types of DNA damage. (a) Schematic of templates with leading-strand DNA damage and the architecture of replication products formed in the absence of leading-strand re-priming. In this and all subsequent figures, red represents leading strands, and blue represents lagging strands; the position of the ARS306 origin of replication is marked, Ori. (b) Replication products from undamaged and damaged (THF, AB, or CPD) templates. (c–d) Two-dimensional gel analysis of the reaction products from undamaged (c) and THF (d) templates at a 60-min time point in (b). See also Supplemental Fig. S1.

We first examined if the fork stalling and uncoupled fork progression we observed in response to a leading-strand CPD [3] were specific to this lesion or were likely to reflect a more general response to blockage of leading-strand replication. To do so, we performed replication reactions using the reconstituted system described previously [3], [15]. Replisomes assembled in this system perform complete leading- and lagging-strand synthesis at the *in vivo* rate with Pol ε synthesizing the leading strand, in conjunction with PCNA and Pol δ on the lagging strand. Maximum rates of fork progression are dependent on the helicase–polymerase coupling factors Csm3/Tof1 and Mrc1 [15]. Reactions were performed on linear templates containing natural or artificial abasic sites or a CPD at a specific site ~ 3 kb left of the origin, ARS306 (Fig. 1a, top left). The natural abasic site (AB) template was prepared by treating a template containing a deoxyuracil residue with uracil DNA glycosylase. The artificial abasic site template contained a more stable synthetic tetrahydrofuran (THF) ring (Supplemental Fig. S1A) [16].

Most products from the undamaged template migrated as full-length products (Fig. 1b, native, lane 1) at an early time point (15 min), containing lagging strands and leftward (8.2 kb) and rightward (1.5 kb) leading strands, visualized in alkaline gels (Fig. 1b, denaturing, lane 1, and Fig. 1c). Note that the lagging-strand maturation machinery was omitted in all the experiments presented herein, and therefore, lagging strands migrate as short Okazaki fragments at the bottom of alkaline gels. In contrast, replication of all three damaged templates led to the accumulation of stalled fork structures (Fig. 1a, top right) after 15 min, detectable as slow-migrating species in native gels (Fig. 1b, native, lanes 4, 7, 10). With time, full-length and uncoupled products (Fig. 1a, bottom left) were produced from the damaged templates (Fig. 1b, native, lanes 6, 9, 12). These products arise due to continued helicase progression to the end of the template, without leading-strand restart but with continued lagging-strand synthesis [3] and were generated with similar kinetics for all three templates. Prominent leading-strand restart products (up to 5.2 kb) were not observed (Fig. 1b, denaturing, lanes 6, 9, 12), confirming restart was rare. Two-dimensional gel analysis verified these assignments: most of the full-length products comprised lagging strands and rightward leading strands, but not leftward leading-strand stall (3 kb) or restart products (Fig. 1d; Supplemental Fig. S1B, C), while uncoupled products contained mostly stalled leading strands.

Some minor damage-specific features were also observed. Reactions performed on AB and THF templates accumulated an additional faster-migrating product in native gels (Fig. 1b, native, “broken forks”). Because the length of these products corresponded to the distance from the damage to the right end of the template (~ 4.5 kb), and they contained mostly 3 kb stalled leading strands, we attribute them to “broken” replication forks, where template breakage at the site of the lesion (either before or after arrival of the fork, or during post-reaction sample processing) generates a double strand break (Fig. 1a, bottom right illustrates one possibility). Consistent with its greater instability, fork breakage was most prominent on the AB template (Fig. 1b, native, compare lanes 6 and 9; Fig. 1d; Supplemental Fig. S1B). In addition, reactions on the THF template contained a small population of full-length (8.2 kb) leading strands from the leftward fork (Fig. 1b, denaturing, lanes 4–6). A portion of these may reflect direct but inefficient synthesis events across the THF by the leading-strand polymerase (Fig. 1a, bottom left), as has been reported for Pol ε in primer extension assays [17], but further work is needed to characterize this in detail.

These experiments demonstrate that two leading-strand template lesions structurally unrelated to a CPD trigger almost indistinguishable responses by the replisome, namely, damage-specific (1) stalled fork formation, (2) delayed progression of forks when leading-strand synthesis and CMG unwinding are uncoupled, and (3) rare leading-strand restart by re-priming. Because all three lesions trigger the replisome to generate structures required for checkpoint activation and damage bypass by template switching (i.e., helicase–polymerase uncoupling with continued lagging-strand synthesis), this may be a default response to any obstacle that specifically blocks leading-strand polymerization. Consistent with this idea, replisome collisions with leading-strand G-quadruplexes are thought to drive uncoupled fork progression in DT40 cells [18].

### Lagging-strand synthesis continues downstream of a leading-strand CPD at all forks

Although the data in Fig. 1 and our previous study [3] showed that lagging-strand synthesis continued at all replication forks that reached the end of a template, an appreciable fraction of stalled replication forks remained unresolved in these experiments for reasons yet to be established. Consequently, the status of lagging-strand synthesis within this population is unclear, and it therefore remains possible that leading-strand damage causes template unwinding to become uncoupled from DNA synthesis at least transiently on both strands, which could perhaps cause persistent fork stalling. Conversely, this would indicate lagging-strand synthesis downstream of damage may be a key driver of uncoupled fork progression. Furthermore, these putative structures would be suboptimal for checkpoint activation and incompatible with damage bypass by template switching.

**Fig. 2:**
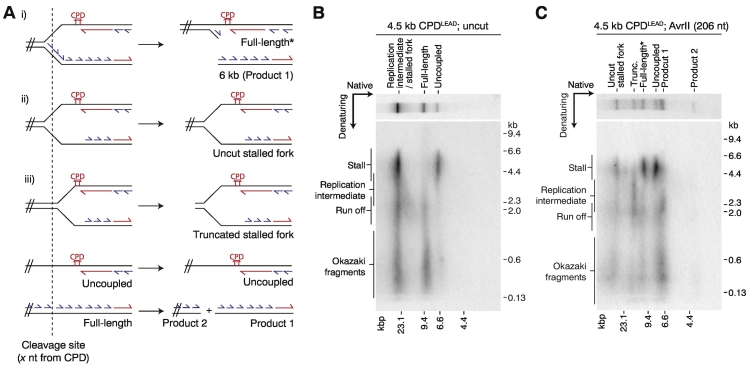
Lagging-strand synthesis continues in the stalled fork population. (a) Schematic showing the predicted digest products for restriction enzyme cleavage of different stalled fork structures (i–iii) and fully resolved products (full length and uncoupled). The full-length* product is generated by single cleavage of a stalled fork in the lagging-strand arm to generate a product that migrates in the position of full length in a native gel. (b and c) Two-dimensional gels showing lagging-strand synthesis downstream of CPD^LEAD^. After AvrII digest (c), stalled forks are depleted and converted to full-length* and product 1 (native dimension), which comprise the expected nascent strands (denaturing dimension) depicted in panel a, top panel. Trunc., truncated replication intermediates. See also Supplemental Fig. S2.

To address this, we devised a strategy to detect lagging-strand synthesis within the population of persistent stalled forks unresolved at the reaction end point. We reasoned that continued lagging-strand replication beyond the damage could be detected by restriction enzyme cleavage using enzymes mapping downstream of the lesion (Fig. 2a and Supplemental Fig. S2A). If lagging-strand synthesis continues within the persistent stalled fork population, forks should be cut once in the lagging-strand arm provided the CMG helicase has unwound the template beyond the enzyme recognition site (Fig. 2a, i). However, if lagging-strand synthesis fails to progress beyond the damage, forks should be resistant to cleavage at sites upstream of the fork junction, as both template strands will be single stranded (Fig. 2b, ii).

To best preserve the architecture of the stalled forks, digests were performed directly in reaction buffer without sample de-proteinization using conditions that supported complete template cleavage in a mock reaction (Supplemental Fig. S2B). The majority of stalled forks (Fig. 2b) were digested by enzymes mapping 82 (Supplemental Fig. S2C), 206 (Fig. 2c), or 374 (Supplemental Fig. S2D) nt downstream of the 4.5-kb CPD^LEAD^. As depicted in Fig. 2a (top panel), cleavage generated full-length* products composed predominantly of stalled leading-strands, and a shorter product (Fig. 2; Supplemental Fig. S2C, D, native, product 1) consisting mainly of Okazaki fragments and rightward leading strands. A minor population of stalled forks were undigested, which could be due to a lack of lagging-strand synthesis, but these could also arise due to failed restriction enzyme cleavage, perhaps due to the presence of RNA primers at the cleavage site. Another minor population (Fig. 2c; Supplemental Fig. S2C, D, native gel, “Trunc.”) migrated between the full-length and stalled forks and contained leftward leading strands that had not yet reached the 4.5-kb CPD^LEAD^. These likely arise from truncation of replication intermediates, which typically comprise 10%–20% of replication products on undamaged templates [3]. Uncoupled products were insensitive to cleavage, confirming that they are single stranded at the restriction sites. Similar cleavage products were also generated by an enzyme mapping 1642 nt beyond the 3-kb CPD^LEAD^ (Supplemental Fig. S2E); however, more of the stalled fork persisted in truncated form (Fig. 2a, iii; Supplemental Fig. S2E, native gel, “Trunc. SF”), suggesting that CMG had not yet reached the restriction site in a sub-population of forks. This supports the view that CMG progression beyond the lesion is heterogeneous; although all CMGs unwind at least ~ 350 bp beyond CPD^LEAD^ [3], some move considerably further despite being visualized as “stalled forks” in native gels. Importantly this is associated in all cases with continued lagging-strand synthesis.

The data in Fig. 2 show that lagging-strand synthesis is maintained efficiently at all replication forks following leading-strand polymerase stalling at a CPD, and therefore compromised lagging-strand synthesis is not the cause of persistent fork stalling. Consequently, all replisomes in our experimental system are capable of forming structures suitable for checkpoint activation and the engagement of template switching DDT pathways. However, given the extent of uncoupled fork progression and hence RPA-ssDNA formation varied greatly between forks within our experiments, the capacity of a given fork for checkpoint activation may also be highly variable. This will be an interesting topic for future work. Finally, these data, together with our previous observation that lagging-strand stalling at a CPD has no detectable influence on leading-strand polymerization rates [3], strongly suggest that leading- and lagging-strand synthesis are uncoupled with respect to each other in the yeast replisome, as is observed in the *Escherichia coli* replisome [19].

### The replisome exhibits an inbuilt brake to slow fork progression following leading-strand polymerase stalling

**Fig. 3:**
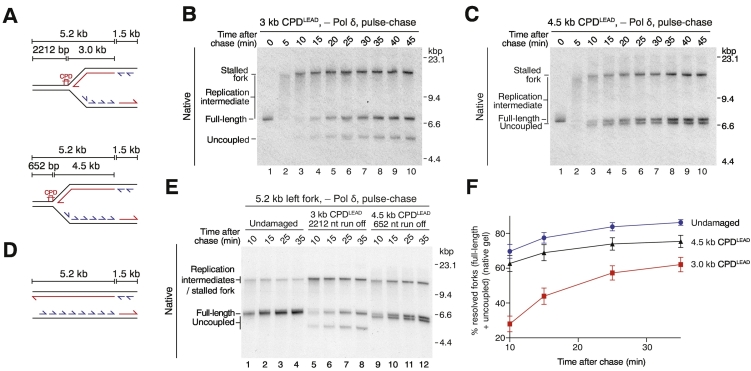
Replisomes slow down after leading-strand polymerase stalling. (a) Schematic of CPD^LEAD^ templates used in this figure. (b and c) Pulse-chase experiments on 3-kb CPD^LEAD^ (b) and 4.5-kb CPD^LEAD^ templates (c). The chase was added at 2 min 50 s. Pol δ was omitted because elevated nucleotide concentrations during the chase promote excessive strand-displacement synthesis. (d) Schematic of undamaged template used in panel e. (e) Pulse-chase experiment performed as in panels b and c. (f) Quantification of experiments performed as in panel e. Error bars represent the SEM from three experiments. See also Supplemental Fig. S3.

We next sought to investigate the dynamics of replication fork progression following leading-strand synthesis blockage. Although the experiments in Fig. 1 clearly show that leading-strand damage delays fork progression, they cannot determine whether the overall reaction kinetics are a consequence of fork stalling for a period of time followed by uncoupled fork progression at the same or similar rates to unperturbed forks, or if uncoupled replication forks generally progress at a much slower rate. To distinguish between these possibilities, we constructed two templates, each with a 5.2-kb leftward fork, but with a leading-strand template CPD (CPD^LEAD^) either 3 or 4.5 kb from the origin (Fig. 3a). In this way, the amount of duplex downstream of the CPD was 2212 or 652 bp, respectively. We then performed pulse-chase experiments to label a population of forks and monitor their progression over time. If forks pause then subsequently resume template unwinding with lagging-strand synthesis at comparable rates to unperturbed forks, both templates should display equivalent resolution kinetics. Conversely, if replisome progression slows down considerably after the damage, the 3-kb CPD^LEAD^ template should accumulate full-length and uncoupled products later than the 4.5-kb CPD^LEAD^ template.

Most replication forks from both damaged templates rapidly reached the CPD and stalled within 10 min of adding the chase (Fig. 3b, c; Supplemental Fig. S3A, B, lane 3). Crucially, resolved daughters (full-length and uncoupled products) accumulated more rapidly from the 4.5-kb CPD^LEAD^ template than the 3-kb CPD^LEAD^ template (Fig. 3b, c, lanes 3–10; Supplemental Fig. S3C). This result demonstrates that uncoupled fork progression proceeds considerably more slowly than fork progression before damage. Additional experiments performed side-by-side with an equivalent undamaged template (Fig. 3d–f; Supplemental Fig. S3D) again revealed a delay in fork resolution for the 3-kb CPD^LEAD^ template relative to the 4.5-kb CPD^LEAD^ template (Fig. 3e). However, the 4.5-kb CPD^LEAD^ template did not exhibit an obvious delay relative to the undamaged template (Fig. 3e, lanes 1, 5, 9, and Fig. 3f). This is likely because the region downstream of the 4.5-kb CPD^LEAD^ was too short (652 bp) to generate an appreciable difference in this assay. For example, even if the template unwinding rate was reduced 10-fold (150–200 bp min^−1^) after polymerase stalling, it would still only take ~ 3–4 min to unwind this distance. Furthermore, we note that due to heterogeneity in the timing of origin firing and the very short pulse used in these experiments, the chase was not 100% efficient, which may have masked more subtle kinetic differences between templates. Finally, the proportion of stalled forks resolved by uncoupled fork progression appears to decrease as the distance beyond the CPD is increased, with full-length and uncoupled products plateauing at a lower value for the 3-kb CPD^LEAD^ than the 4.5-kb template. This result may indicate that the processivity of replication forks is reduced downstream of DNA damage in this system, which could give rise to the population of persistent stalled forks that we consistently observe.

Collectively, these data reveal a replisome-intrinsic mechanism to considerably slow replication fork progression in response to leading-strand template damage while maintaining robust lagging-strand synthesis. Our data are consistent with a recent report showing CMG slowing following bypass a leading-strand DNA-protein crosslink in *Xenopus* egg extracts. Importantly, this study did not exclude roles for the checkpoint, DNA repair pathways or chromatin-mediated effects in this phenomenon [20], and our work therefore extends these findings by demonstrating that fork slowing is intrinsic to the replisome. This mechanism should help to ensure that appropriate DNA structures are generated for checkpoint activation while limiting excessive production of ssDNA on the leading strand. Slow uncoupled fork progression may also be important to enable DDT pathways to engage with forks for lesion bypass before excessively long tracts of ssDNA have been generated. Furthermore, should the impeded replication fork be rescued by a fork from a neighboring origin, the ssDNA gap between the DNA lesion and site of fork convergence will be kept to a minimum, which should help facilitate efficient post-replicative ssDNA gap filling.

### Fork slowing and stalling are triggered by a cessation of leading-strand synthesis

**Fig. 4:**
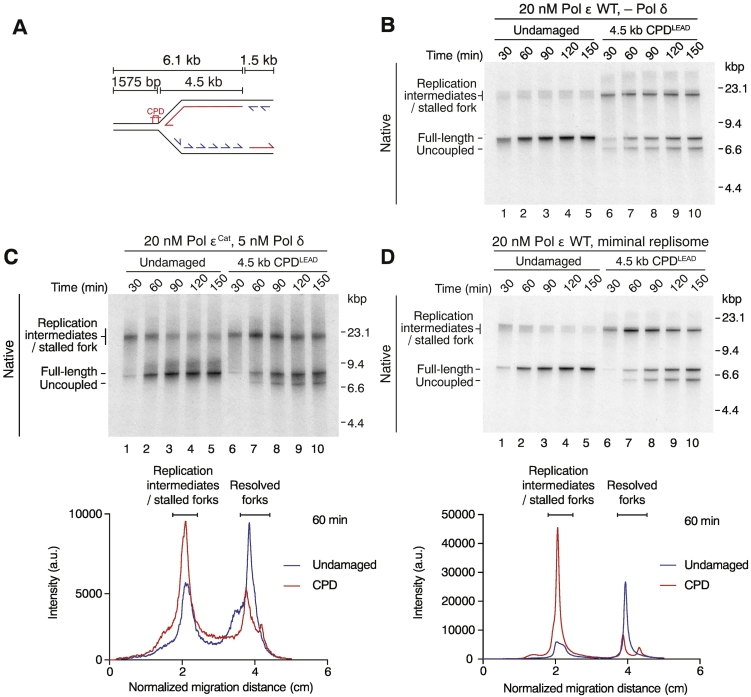
Cessation of leading-strand synthesis is a key mechanism underlying fork stalling and slowing. (a) Schematic of CPD^LEAD^ templates used in panels b–d. (b and c) Replication reaction on undamaged and damaged templates in the absence of Pol δ (b) or in the presence of Pol δ and Pol ε^Cat^ (c). Reactions contained 267 mM potassium glutamate, which prevents leading-strand synthesis by Pol α [15]). Lane profiles of replication products at 60 min are shown for the experiment in panel c. (d) Replication reactions performed on undamaged and damaged templates using the minimal replisome described in Ref. [15] but lacking Ctf4. Lane profiles of replication products at 60 min are shown. See also Supplemental Fig. S4.

The CMG helicase and Pol ε are intimately associated physically and functionally in executing rapid and efficient leading-strand synthesis [15], [21], [22], [23], [24]. Therefore, we considered the possibility that catalysis of leading-strand synthesis specifically by Pol ε prior to the lesion may be important for fork stalling and slowing (e.g., if the stalled catalytic domain of Pol ε remains at the lesion, creating a Pol ε-specific “tether” that restricts CMG progression). To test this, we enforced leading-strand synthesis by Pol δ using a catalytic mutant of Pol ε (Pol ε^Cat^) competent for origin firing but not DNA polymerase activity [25] on a template with 1575 bp beyond CPD^LEAD^ (Fig. 4a). Control experiments with Pol ε but without Pol δ (Fig. 4b; Supplemental Fig. S4A) yielded similar results to those in Fig. 1. As reported [15], [25], replisomes established in the absence of Pol ε catalytic activity displayed greatly reduced fork progression even in the absence of damage, accounting for many products existing as replication intermediates after 30 min (Fig. 4c; Supplemental Fig. S4B; lane 1). However, over time the majority of these intermediates resolved to completely replicated daughters (Fig. 4c; Supplemental Fig. S4B; lanes 2–5). We observed greater heterogeneity between replication reactions performed with Pol ε^Cat^ on undamaged templates, as judged by the ratio of replication intermediates to full-length products over the same time course in identical reactions (Fig. 4c; Supplemental Fig. S4C, D, native; lanes 1–5). We do not fully understand the reasons for this, but it may reflect variability in elongation rates given the reported poor functionality of Pol δ with CMG [15], [21]. Nevertheless, we observed damage-dependent delays in replication fork progression in multiple experiments under these conditions (Fig. 4c; Supplemental Fig. S4C, D; compare lanes 7–10 with 2–5), confirmed by quantification of the average extent of fork resolution on damaged relative to undamaged templates (Supplemental Fig. S4E). Catalysis of leading-strand replication by Pol ε before the replisome encounters a lesion in this strand is therefore not required for prolonged fork stalling and delayed replication fork progression.

Because structurally distinct leading-strand lesions delayed fork progression to a similar extent (Fig. 1) and catalysis by Pol ε is not required to observe this delay on a CPD template (Fig. 4a–c), we considered that it may be a direct consequence of the cessation of leading-strand polymerization behind the CMG helicase. We therefore sought to test this hypothesis in the most simplistic context by including only those proteins required for CMG assembly/activation and the initiation and maintenance of leading-strand synthesis [26]. The minimal replisome assembled under such conditions is capable of replicating large plasmid templates (10 kb) but does so at greatly reduced rates due to the absence of Mrc1 and Csm3/Tof1 [15], [26]. Despite the slow rates of fork progression, full-length products were the major reaction species generated by the minimal replisome after 60 min on an undamaged template (Fig. 4d; Supplemental Fig. 4F). In contrast, a significant fraction of replication forks remained stalled after 60 min on the 4.5-kb CPD^LEAD^ template. As the reaction progressed, the stalled fork population declined and there was a concomitant increase in uncoupled and full-length products. However, similar to our earlier experiments, a population of stalled forks remained unresolved at the longest time point (150 min). These data therefore reveal that the minimal replisome responds to leading-strand lesions in much the same way as the more complete replisome used in Fig. 1, Fig. 2, Fig. 3 and when Pol δ is synthesizing the leading strand (Fig. 4a–c).

We propose that replication fork stalling and slow uncoupled fork progression are direct consequences of the cessation of leading-strand polymerization downstream of a blockage on this strand, with the simplest model being that the rate of fork progression defaults to the template unwinding rate of the isolated CMG helicase. This is further supported by our previously published observation that restoring leading-strand synthesis by addition of an oligonucleotide mapping downstream of CPD^LEAD^ increases fork progression rates [3]. It is not possible to precisely measure rates of uncoupled fork progression from our experiments, and therefore we cannot yet determine if they are influenced by factors such as Csm3/Tof1/Mrc1, which are crucial for normal rates of fork progression [15], or Pol ε in a non-catalytic capacity. Single-molecule experiments with labeled replication proteins will likely be required to address this. Furthermore, given the roles of Csm3/Tof1/Mrc1 in checkpoint activation/amplification [27] and control of uncoupling in response to hydroxyurea [14], it is possible that these proteins act to further modulate uncoupled fork progression in response to leading-stand damage *via* a checkpoint-dependent pathway. Greater insights into the structures and mechanisms of these proteins at replication forks will help to address these questions.

In addition to providing further insights into the earliest response of the eukaryotic replisome to leading-strand damage, our experiments provide more evidence of a functional role for leading-strand polymerization in driving DNA unwinding by CMG at speeds above and beyond those intrinsic to the isolated enzyme complex. While this had been proposed for Pol ε in conjunction with Csm3/Tof1/Mrc1 [15], our work now indicates more generally that the act of leading-strand polymerization can increase template unwinding rates. Further work is required to elucidate the mechanism by which template unwinding is stimulated by leading-strand polymerization, although one possibility is that the presence of the nascent leading-strand helps prevent backtracking of the CMG helicase. Interestingly, despite the prokaryotic and eukaryotic replicative helicases translocating on different template strands, replisomes from bacteriophage T7 [28] and bacteria [29] also achieve maximum rates by coupling leading-strand synthesis to template unwinding. Furthermore, helicase slowing occurs following stochastic leading-stand polymerase pausing in the *E. coli* replisome during unperturbed fork progression, which is important to ensure complete replication without coordination of leading- and lagging-strand synthesis [19]. The strong dependence on leading-strand polymerization for maximum unwinding rates at replication forks therefore represents a conserved coupling mechanism that provides an autonomous failsafe, akin to a “dead man's switch” [19], to limit rapid and deleterious ssDNA exposure following interruptions to leading-strand synthesis.

## Materials and Methods

### Proteins

All proteins were purified as described previously [3], [15], [26], [30]. Pol ε^Cat^ contains the polymerase active site mutation Pol2-D640A.

### Replication assays and templates

Plasmids with and without DNA damage were prepared as described previously [3] by insertion of oligonucleotides into cassettes at specific sites either ~ 3 kb (plasmid ZN3) or 4.5 kb (plasmid ZN5Sp) from the origin using the following oligonucleotides: undamaged DNA: 5′-phos-TCAGCACTTAAGTCC; THF: 5′-phos-TCAGCACT-/idSp/-AAGTCC, CPD: 5′-phos-TCAGCAC-/CPD/-AAGTCC. To generate replication assay templates, plasmids were all linearized with AhdI as previously described [3], and in the following cases additionally with AsiSI (Fig. 3 and Supplemental Fig. S3) and PsiI (Fig. 4 and Supplemental Fig. S4). The AB template was prepared by inserting an oligonucleotide containing deoxyuracil (5′phos-TCGCACT/ideoxyU/AAGTCC), and after linearization with AhdI, the template was treated with UDG (NEB M0280) in the same buffer for 1 h. In Fig. 4, the undamaged template was prepared by linearizing Maxi prep DNA.

Standard replication assays were performed exactly as described in detail previously [3]. Briefly, MCM2–7 loading was performed at 24 °C for 10 min, and S-CDK was added for a further 5 min before the reaction was initiated and transferred to 30 °C. The final reaction buffer composition (except where indicated) was as follows: 29.2 mM Hepes–KOH (pH 7.6), 217 mM potassium glutamate (except Fig. 3, Fig. 4d; Supplemental Figs. S3, S4F: 117 mM), 0.0117% NP-40-S, 1.17 mM DTT, 11.7 mM Mg(OAc)_2_, 0.117 mg/ml BSA, 6.7 mM KCl, 3 mM ATP, 400 μM CTP, GTP, UTP, 30 μM dATP, dCTP, dGTP, dTTP, 33 nM α-[^32^P]-dCTP, 12.5 nM Cdt1/Mcm2–7, 7.5 nM Cdc6, 3.3 nM ORC, 8.3 nM DDK, 20 nM S-CDK, 30 nM Dpb11, 210 nM GINS, 40 nM Cdc45, 20 nM Pol ε, 5 nM Mcm10, 20 nM Ctf4, 60 nM RPA, 20 nM Csm3/Tof1, 20 nM Mrc1, 20 nM RFC, 20 nM PCNA, 10 nM TopoI, 20 nM Pol α, 5 nM Pol δ, 25 nM Sld3/7, and 50 nM Sld2. In pulse-chase experiments in Fig. 3 and Supplemental Fig. S3, Pol δ was omitted, and the reaction was initiated at a lower dCTP concentration (2.5 μM), and after 2 min 50 s, the concentration of all four dNTPs increased to 400 μM. Reactions were quenched with 25–30 mM EDTA except in Fig. 2 and Supplemental Fig. S2, where after 90 min the reaction mix was diluted 4-fold in 25 mM Hepes–KOH (pH 7.6), 10 mM Mg(OAc)_2_, and 60 nM RPA before the addition of water (negative control), PmeI, AvrII, or AfeI (NEB) (3 μl enzyme in 56 μl diluted reaction mix) and incubation at 37 °C for 10 min, followed by quenching with EDTA.

Following quenching, proteins were removed with 0.1% SDS and 1/100 volumes proteinase K (NEB P8107) at 37 °C for 20 min, and the DNA was extracted with phenol–chloroform. Samples were passed over illustra MicroSpin G-50 columns (GE Healthcare). Where indicated in figures, samples for the denaturing gel were digested with SmaI. Samples were resolved in native and alkaline (denaturing) agarose gels and visualized by autoradiography or phosphorimaging as described in detail previously [3].

### Data analysis

Extraction of lane profiles and replication product quantification was performed in ImageJ after converting the .gel files to 16-Bit Tiff files using the Linearize GelData command.

## Appendix A Supplementary data

Supplementary figures Image 1
