# Supplementary material for: Dynamics of Replication Fork Progression Following Helicase–Polymerase Uncoupling in Eukaryotes
Source: J Mol Biol. 2019 May 3;431(10):2040–9. doi: 10.1016/j.jmb.2019.03.011 (PMC6525111; doi:10.1016/j.jmb.2019.03.011)
Supplement: Supplementary file 1 — Supplementary figures [file mmc1.pdf]

Supplemental Figure S1

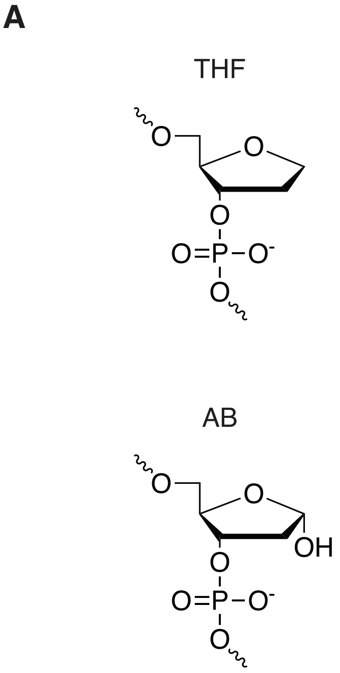

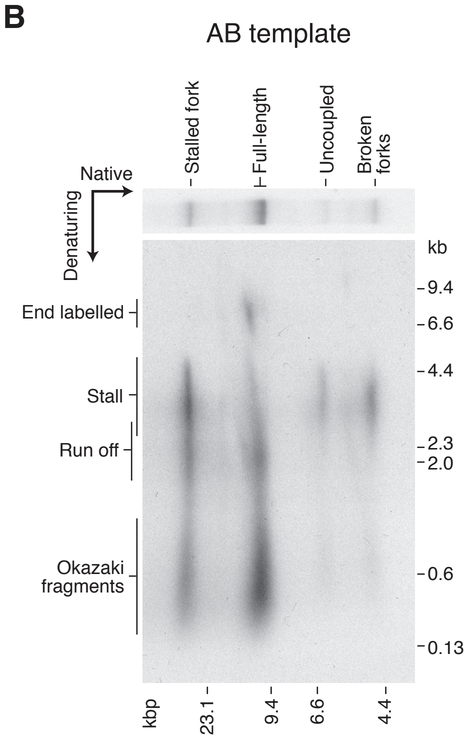

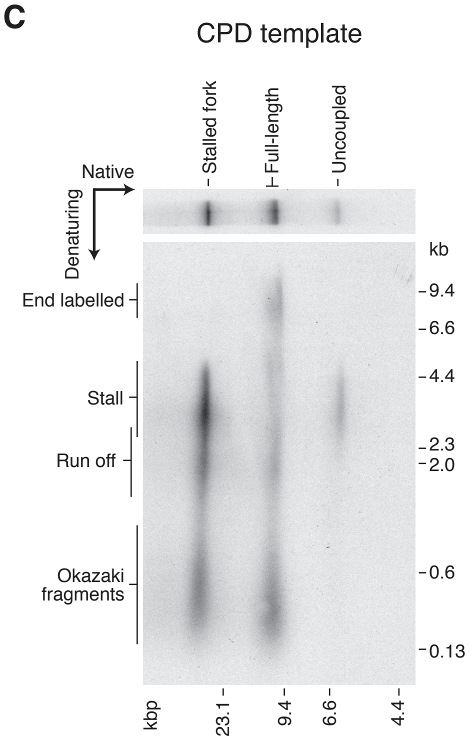

Supplemental Figure S1

(A) Chemical structures of THF and AB lesions in the context of DNA backbone. The THF lesion lacks the 1' position hydroxyl group naturally generated by DNA glycosylases, which improves its chemical stability relative to the AB template (Takeshita et al. 1987).

(B-C) Two dimensional gel analysis of the nascent strand composition of native gel products from AB (B) and CPD (C) templates at a 60 min time point in Fig. 1B.

Supplemental Figure S2

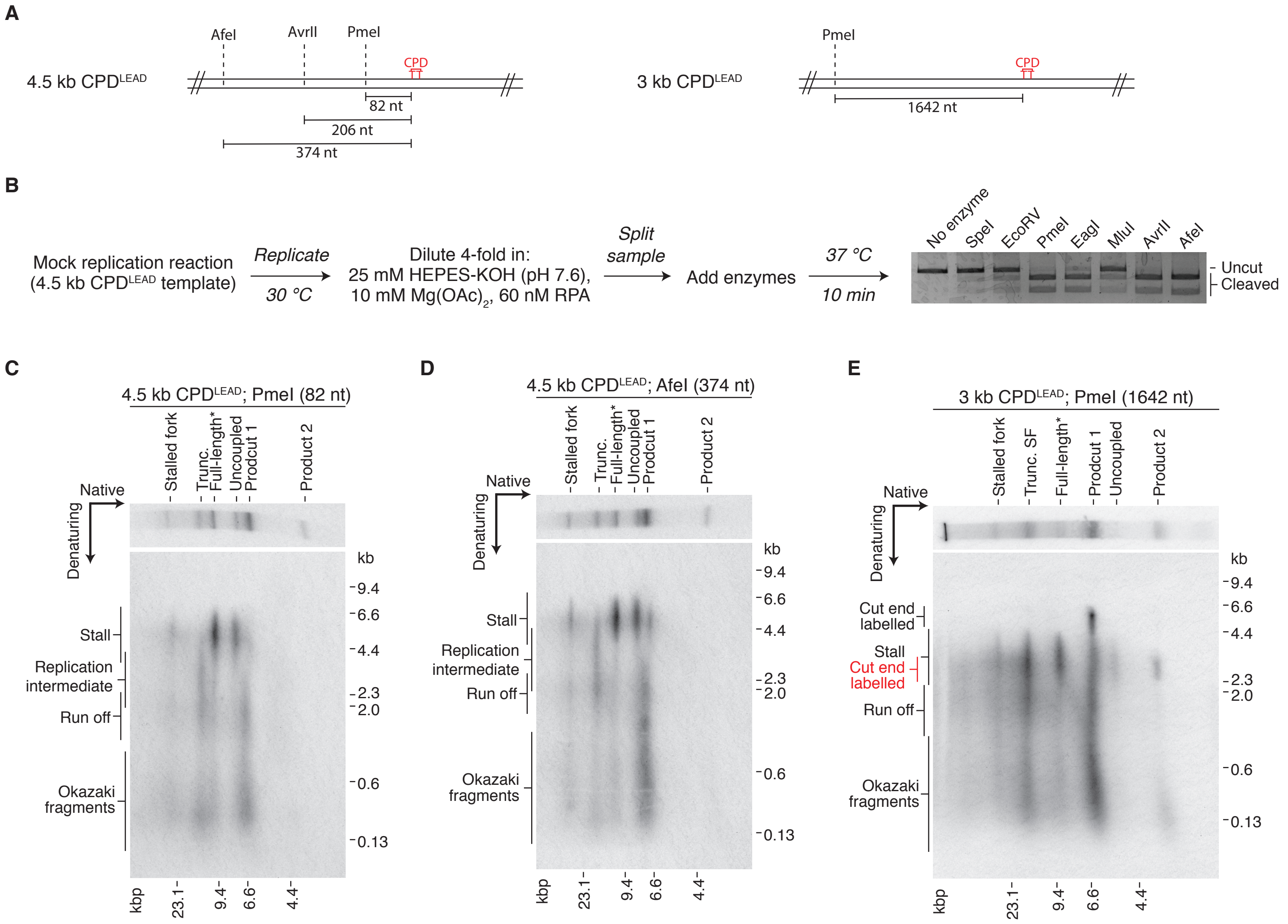

Supplemental Figure S2

(A) Diagrams of the 4.5 kb and 3 kb CPD^{LEAD} templates illustrating the locations of restriction enzyme sites used for stalled fork mapping.

(B) Schematic of experiment and ethidium-bromide stained agarose gel showing DNA products of treatment of a mock replication reaction with the indicated restriction enzymes.

(C and D) Two-dimensional gels showing lagging-strand synthesis downstream of CPD^{LEAD}. Samples were taken from the same experiment after 90 min as in Figure 4 but digested for 10 min with PmeI (C) or AfeI (D) instead. After digestion, stalled-forks are depleted and converted to full-length* and product 1 in the native gel, which comprise the expected nascent strands depicted in Fig. 2A, panel i), in the denaturing dimension. Trunc.: truncated replication intermediates.

(E) Two-dimensional gel showing sub-populations of stalled forks with different extents of CMG unwinding downstream of CPD^{LEAD}. After 90 min, reaction products were digested for 10 min with PmeI. Some stalled forks are converted to truncated stalled forks (Trunc. SF), as depicted in Fig. 2A, panel iii), indicating CMG had not reached the PmeI site, whereas others are converted to similar products as in Fig. 2C and Supplemental Figs. S2C,D, indicating CMG unwinding and lagging-strand synthesis had continued past the PmeI site.

Supplemental Figure S3

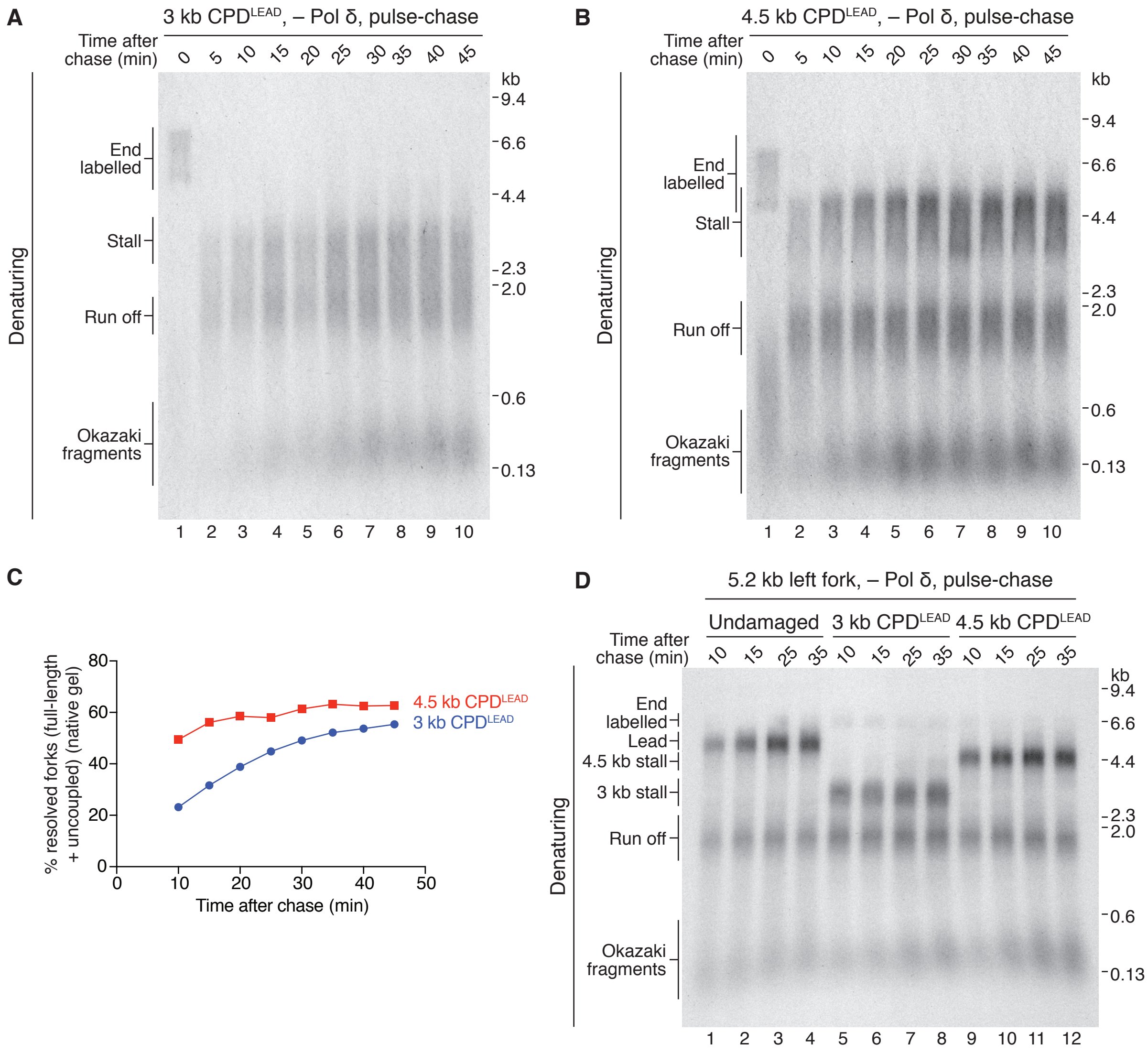

Supplemental Figure S3

(A and B) Denaturing gels corresponding to the experiments in Figs. 3B (A) and 3C (B)

(C) Quantification of data presented in Fig. 3B and C.

(D) Denaturing gel corresponding to the experiment in Fig. 3E.

Supplemental Figure S4

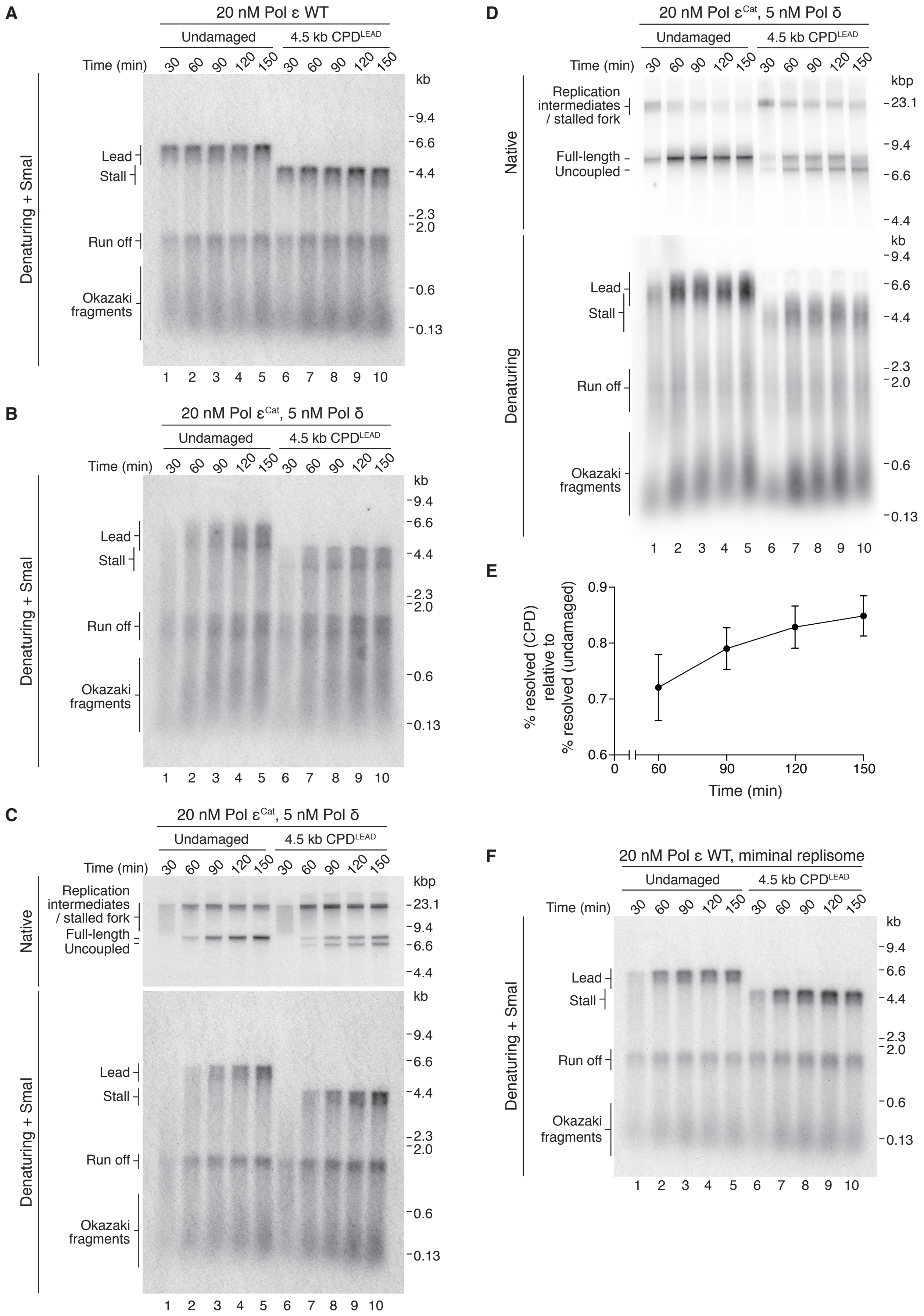

Supplemental Figure S4

(A) Denaturing gel corresponding to the experiment in Fig. 4B.

(B) Denaturing gel corresponding to the experiment in Fig. 4C.

(C and D) Independent experimental repeats performed in the same way as Fig. 4C and Supplemental Fig. S4B, showing replication products from undamaged and damaged templates (potassium glutamate: 267 mM) in the presence of Pol δ and Pol ε^{Cat}.

(E) Quantification of the data in Fig. 4C (native) and Supplemental Figs. S4C,D (native). The relative extent of resolution was calculated by dividing the percentage of resolved forks (sum of the full-length and uncoupled products) from the damaged template by the same percentage from the undamaged template at each time point from 60 to 150 minutes. The mean relative resolution was then calculated. Error bars represent the SEM from three experiments.

(F) Denaturing gel corresponding to the experiment in Fig. 4D.
